# Kaempferol, Myricetin and Fisetin in Prostate and Bladder Cancer: A Systematic Review of the Literature

**DOI:** 10.3390/nu13113750

**Published:** 2021-10-23

**Authors:** Felice Crocetto, Erika di Zazzo, Carlo Buonerba, Achille Aveta, Savio Domenico Pandolfo, Biagio Barone, Francesco Trama, Vincenzo Francesco Caputo, Luca Scafuri, Matteo Ferro, Vincenzo Cosimato, Ferdinando Fusco, Ciro Imbimbo, Giuseppe Di Lorenzo

**Affiliations:** 1 Urology Unit, Department of Neurosciences, Reproductive Sciences and Odontostomatology, University of Naples “Federico II”, 80121 Naples, Italy; achille-aveta@hotmail.it (A.A.); pandolfosavio@gmail.com (S.D.P.); biagio.barone@unina.it (B.B.); vincitor@me.com (V.F.C.); ciro.imbimbo@unina.it (C.I.); 2 Department of Precision Medicine, University of Campania “Luigi Vanvitelli”, 80138 Naples, Italy; erika.dizazzo@unimol.it; 3 Department of Medicine and Health Sciences “V. Tiberio”, University of Molise, 86100 Campobasso, Italy; direttoreuocpagani@gmail.com; 4 Oncology Unit, Hospital ‘Andrea Tortora’, ASL Salerno, 84016 Pagani, Italy; lucaluca@hotmail.it; 5 Associazione O.R.A., Somma Vesuviana, 80049 Naples, Italy; 6 Andrology and Urogynecology Clinic, Santa Maria Terni Hospital, University of Perugia, 05100 Terni, Italy; francescotrama@gmail.com; 7 Division of Urology, European Institute of Oncology IRCCS, 20141 Milan, Italy; matteo.ferro@ieo.it; 8 Division of Laboratory Medicine, Civil Hospital ‘Maria SS. Addolorata’, ASL Salerno, 84025 Eboli, Italy; cosimatov@gmail.com; 9 Urology Unit, Department of Woman Child and of General and Specialist Surgery, University of Campania “Luigi Vanvitelli”, Via S. Pansini 5, 80131 Naples, Italy; ferdinando.fusco@unicampania.it

**Keywords:** fisetin, kaempferol, myricetin, prostate cancer, bladder cancer

## Abstract

Prostate and bladder cancer represent the two most frequently diagnosed genito-urinary malignancies. Diet has been implicated in both prostate and bladder cancer. Given their prolonged latency and high prevalence rates, both prostate and bladder cancer represent attractive candidates for dietary preventive measures, including the use of nutritional supplements. Flavonols, a class of flavonoids, are commonly found in fruit and vegetables and are known for their protective effect against diabetes and cardiovascular diseases. Furthermore, a higher dietary intake of flavonols was associated with a lower risk of both bladder and prostate cancer in epidemiological studies. In this systematic review, we gathered all available evidence supporting the anti-cancer potential of selected flavonols (kaempferol, fisetin and myricetin) against bladder and prostate cancer. A total of 21, 15 and 7 pre-clinical articles on bladder or prostate cancer reporting on kaempferol, fisetin and myricetin, respectively, were found, while more limited evidence was available from animal models and epidemiological studies or clinical trials. In conclusion, the available evidence supports the potential use of these flavonols in prostate and bladder cancer, with a low expected toxicity, thus providing the rationale for clinical trials that explore dosing, settings for clinical use as well as their use in combination with other pharmacological and non-pharmacological interventions.

## 1. Introduction

Prostate and bladder cancer represent the two most frequently diagnosed genito-urinary malignancies, with 1,414,259 and 573,278 cases estimated to have been diagnosed in 2020 [1]. In spite of considerable advances in the field of diagnosis and treatment [2,3,4], mortality remains high, with an estimated 375,304 and 212,536 people dying because of prostate and bladder cancer, respectively, in 2020 [1]. Population-based screening has proven to be useful for early detection of prostate cancer [5], while its benefits remain unproven in bladder cancer [6]. Primary prevention interventions aimed at preventing the onset of the disease through action on modifiable risk factors have true potential for reducing prostate and bladder cancer mortality. The most commonly known risk factors for prostate cancer include age, lifestyle, sexual habits, family history, ethnicity as well as occupational and environmental exposure [7,8,9], while risk factors for bladder cancer include smoking, age, gender, occupational and environmental exposure as well as infection with Schistosoma haematobium [10,11]. Diet may affect both prostate [12] and bladder [13] cancer risk. While highly processed foods are associated with a higher prostate cancer risk, soy, lycopene-rich foods and fish may exert a protective effect [14]. Bladder cancer risk may be increased by higher intakes of processed meat, while it may be decreased by higher consumption of fruit, vegetables, citrus fruit, and cruciferous vegetables [13]. Dietary preventive measures, including the use of nutritional supplements, might therefore be part of a preventive strategy against both prostate and bladder cancer.

Flavonoids represent a class of polyphenolic compounds that are normally present in the human diet [15]. The chemical structure of flavonoids presents a benzene ring that is condensed with a 6-member ring and has a phenyl ring attached either to the C2 or the C3 carbon position [16]. Several classes of flavonoids have been identified based on their chemical structure. Among these, flavonols, which are characterized by a distinctive hydroxyl group at the C3 carbon position [17], represent the most ubiquitous flavonoids present in food [18]. Flavonols have been extensively investigated during the past decades as they have been convincingly associated with favourable biological activities, including a protective effect against diabetes [19] and cardiovascular diseases [20]. Furthermore, a higher dietary intake of flavonols was reported to be related to a lower risk of both bladder [21] and prostate [22] cancer. Among the several known flavonols, quercetin and its glycosylated form, isoquercetin represent the most studied compounds [23]. Isoquercetin has also been tested as a GMP medicinal product in prospective clinical trials as an adjunct therapy against sunitinib-induced fatigue by Buonerba et al. [24] and as preventive measure against cancer-associated thrombosis by Zwicker et al. [25]. Other less extensively studied—yet also promising—flavonols include kaempferol, fisetin and myricetin. Kaempferol is a naturally occurring flavonol that can be found in tea as well as in grapefruit, beans, apples, kale, brussel sprouts, cabbage, grapes, broccoli, tomatoes, citrus fruits, gooseberries and strawberries [26]. Kaempferol has displayed strong anti-inflammatory, anti-neoplastic, cardio- and neuro-protective properties in a number of pre-clinical studies, with no expected toxic effects in humans [27]. Fisetin is also found in vegetables and fruits, such as cucumber, persimmon apple, grape, onion, and strawberry [28]. Besides showing antioxidant, anti-inflammatory and antiproliferative activity, fisetin may display a peculiar capacity to target senescent cells, which are resistant to apoptosis and are associated with chronic diseases and aging [29]. Finally, myricetin can also be isolated in several plant families and is commonly found in fruits and vegetables [30]. Similarly to other flavonols, myricetin has shown multiple attractive properties, including antibacterial, antiviral, anti-inflammatory, anti-tumor, anti-obesity, cholesterol-lowering effects, along with cardio- and neuro-protective activity [31].

We here present a narrative review based on a systematic search of the literature aimed at assessing currently available evidence regarding the anti-neoplastic effect of kaempferol, fisetin and myricetin in prostate and bladder cancer. Potential applications are also discussed from a multidisciplinary (urologist/oncologist/nutritional biologist) perspective.

## 2. Materials and Methods

The systematic review was conducted following PRISMA principles, where applicable [32]. PUBMED, SCOPUS and EMBASE were used for the systematic review of the literature. The search string included the following terms: “prostate cancer”, “bladder cancer”, “kaempferol”, “myricetin”, “fisetin”. No temporal limits were applied. Original articles reporting in vitro, animal and human studies were included. Non-original articles (editorials, review articles, letters to the editor, etc) as well as articles describing purely theoretical models or reporting chemical/pharmacological experiments were excluded. The systematic review was conducted by FC and CB in August 2021. Any discrepancies were resolved through a consensus discussion with a third author (VC). The number of included and excluded articles is reported in Table 1.

## 3. Results of the Systematic Review

### 3.1. Kaempferol

#### 3.1.1. Preclinical Studies

Kaempferol has shown anti-neoplastic activity in multiple pre-clinical models of prostate and bladder cancer. In a pre-clinical study employing 3-(4,5-dimethylthiazol-2-yl)-2,5-diphenyltetrazolium bromide (MTT) assay to assess cell viability [33], kaempferol at concentrations of 5, 10 and 15 μM yielded a reduction in androgen-dependant LNCaP cells growth of 33%, 60% and nearly 100%, respectively. Another pre-clinical study based on the trypan blue cell counting assay reported a half maximal effective concentration (EC_50_) for kaempferol of 38.35 ± 1.94 and 33.29 ± 2.96 μM in androgen-independent DU-145 and PC-3 cells, respectively. Importantly, at these concentrations, kaempferol had no impact on the viability of human foreskin fibroblasts (HFF) cells, which provides evidence supporting a favourable efficacy/toxicity profile for kaempferol [34]. Kaempferol anti-neoplastic activity was confirmed in another in vitro study using a WST-1 cell viability assay, which showed that 10 μM kaempferol reduced cell proliferation by 20% in LNCaP cells [35]. Another pre-clinical study [36] conducted in DU-145 cell culture reported that 50 μM kaempferol was associated with a 50% growth rate reduction in MTT assay. One preclinical study that used the SRB (Sulforhodamine B) assay showed that kaempferol displayed potent cytotoxic activity towards several cancer cell lines (including PC-3), with IC_50_ values in the 1.0–2.3 μM range [37]. Kaempferol has also shown potent activity in bladder cancer pre-clinical models. One study reported a 50–58% reduction in EJ cell viability after exposure to 20–54.7 μM kaempferol, with no effect on the growth of normal bladder cells SV-HUC-1 exposed to 10–40 μM kaempferol [38]. In a mouse model developed by injecting bladder cancer cells subcutaneously into nude mice, kaempferol injected intraperitoneally at a dose of 50–150 mg/kg daily for 4 weeks was associated with a tumor weight reduction in the range of 30–60%, measured after sacrificing the animal with no apparent toxicity [39]. Furthermore, immunohistochemistry analysis (TUNEL assay) of cancer tissues showed that in mice treated with 150 mg/kg kaempferol, a 70% apoptotic rate was detected compared to 7% in control mice, with decreased expression of c-Met, cyclin B1, and c-Fos [39]. Hypothesised mechanisms of action of kaempferol anti-neoplastic activity involve blocking the cell cycle progression [40], induction of apoptosis [39], inhibition of Anoctamin 1 (ANO1), a calcium-activated chloride channel [41], inactivation of oncogenic proline-directed protein kinase FA [42], which is involved in neoplastic transformation and progression, inhibition of cyclooxygenase-2 [43], inhibition of fatty acid synthase activity [44], inhibition of Glyoxalase 1 [45], increased synthesis of granulocyte-macrophage colony-stimulating factor [46], modulation of DNA methylation [47], and induction of oncosuppressor protein PTEN [48].

#### 3.1.2. Clinical Studies

Only limited evidence is available from epidemiological studies that have investigated the potential association between dietary consumption of kaempferol and prostate and bladder cancer. In a case-control study conducted in western New York including 433 men with histologically confirmed prostate cancer and 538 population-based controls, matched according to age and county of residence, a non-statistically significant 10–20% reduction in the odds of having prostate cancer was found for those who consumed more kaempferol [49]. It must be considered that overall intake of kaempferol was low (approximately 20 mg/a day) both in cancer cases and controls, compared to doses required to achieve serum concentrations investigated in preclinical models. Conversely, in an epidemiological study based on the Netherlands Cohort Study involving 3362 men with prostate cancer, of whom 1164 men with advanced disease, who were followed-up for a period of 17.3 years, a higher intake of kaempferol was associated with a significantly decreased hazard ratio of advanced prostate cancer [50]. One study reviewing data from a Spanish case-control study was conducted to explore the association between bladder cancer and specific carotenoids (alpha-carotene, lutein, lycopene and beta-carotene) and flavonoids (quercetin, kaempferol, myricetin, and luteolin). In the analyzed population, which included 497 newly diagnosed bladder cancer and 1113 matched controls, neither total intake of carotenoids/flavonoids nor specific compounds (including kaempferol) were associated with bladder cancer [51]. Although the study has merit in its investigation of the potential relationship of bladder cancer with specific flavonoids, its small sample size is a major limitation that underlines the need for larger epidemiologic studies.

### 3.2. Fisetin

#### Preclinical Studies

Fisetin anti-neoplastic activity has also been assessed in multiple pre-clinical prostate and bladder cancer models. In a cellular model that used fisetin as a positive control, the IC_50_ of fisetin was 34.1 ± 7.7 μM as measured by WST-1 cells in LNCaP cell culture [52]. In mice, fisetin (1 mg/kg) intraperitoneally daily) significantly reduced both the tumor weight and size of the xenograft prostate tumors [53]. In another preclinical study, fisetin (10–60 μM) was associated with decreased cell viability in LNCaP cells (19–62%) and CWR22Rυ1 cells (18–55%) after 48 h treatment, with minimal effect on prostate epithelial cells at the same concentrations. Furthermore, this study showed that in nude mice bearing xenograft prostate cancer tumors, fisetin (1 mg/day intraperitoneally) was associated with an average tumor volume of 302 mm^3^ after 26 days treatment, compared to an average tumor volume of 1200 mm^3^ in controls [54]. Fisetin (20 μM) has also been found to synergize with cabazitaxel (5 μM) in cellular models of 22Rν1, PC-3M-luc-6, and C4-2 prostate cancer cell lines [55]. In these three cell lines, a 45%, 49% and 74% decreased cell viability, respectively, was reported with fisetin alone while a 32%, 11% and 38% decreased cell viability was reported with cabazitaxel alone. This model showed that fisetin can synergize with cabazitaxel, as a combination of fisetin plus cabazitaxel yielded a reduction in cell viability by approximately 79%, 53% and 78%, respectively, in the three cell lines assessed. In nude mice bearing prostate cancer xenografts, the authors explored the effect of intraperitoneal injection of either fisetin (20 mg/kg; 3 times/week) alone, cabazitaxel (5 mg/kg; once/week) alone, fisetin (20 mg/kg; 3 times/week) plus cabazitaxel (5 mg/kg; once/week), or vehicle. Of note, while fisetin alone and cabazitaxel alone were associated with 22% and 31% inhibition of tumor growth, respectively, cabazitaxel plus fisetin yielded a 53% inhibition of tumor growth compared to the control group [55].

One of the possible mechanisms of action of fisetin may be mediated by decreased synthesis of ialuronic acid [56], as higher levels of ialuronic acid in the tumor microenvironment are associated with prostate cancer progression [57]. Additional putative mechanisms of action of fisetin in prostate cancer include microtubule stabilization (10 μM) [58], inhibition of epithelial-to-mesenchymal transition (60 μM) via inhibition of YB-1 [53], TRAIL-mediated augmentation of apoptosis (50 μM) [59], inhibition of cell cycle (1–50 μM) [60,61,62], induction of autophagic cell death via mTOR suppression [63], and inactivation of the JNK and PI3K/Akt signaling pathways [64]. Fisetin may also favorably modulate gut microbiota [65], which is possibly involved in prostate cancer etiopathogenesis [66].

Fewer studies have been conducted in bladder cancer models, although fisetin has also shown promising results as an anti-neoplastic agent in this tumor. In a bladder cancer cellular model, 60 μM fisetin was associated with an approximately 60% cell viability after 48 h in T24, EJ, J82 cell lines [67]. In a rat model of bladder cancer induced by intravesical N-methyl-N-nitrosourea (MNU) [68], fisetin + MNU yielded tumor occurrence in 22.2% of rates (4/18) compared to 70.6% (12/17) of MNU alone. This finding is consistent with the results obtained in another study showing that fisetin was moderately inhibitory to mutagenicity associated with benzidine, a human bladder carcinogen, in the Ames Salmonella microsome/mutagenicity assay [69], which provides proof of concept evidence supporting the use of fisetin as a preventive bladder cancer agent. Putative biological mechanisms of fisetin anti-neoplastic activity in bladder cancer involve apoptosis and cell cycle arrest via activation of p53 and inhibition of NF-kappa B pathway [67,68].

### 3.3. Myricetin

#### 3.3.1. Preclinical Studies

Myricetin has shown promising antineoplastic activity in a few pre-clinical models of prostate and bladder cancer. In a cellular model employing multiple prostate cancer cell lines, myricetin IC_50_ values measured via CCK-8 and colony formation assays were 47.6 µM, 55.3 µM, 79.9 µM in prostate cancer cell lines PC3, DU145, C4-2, respectively, while it was much higher (362.1 µM) in normal epithelial prostate cell line RWPE-1 [70]. In nude mice, myricetin (25 mg/kg) administered every other day by intraperitoneal injection was able to induce regression of PC3 subcutaneous xenografts compared to controls, with an average tumor volume three times lower in myricetin-treated mice compared to control on day 45. Myricetin was able to induce apoptosis in PC3 cells, as shown by flow cytometry, with increased expression levels of the apoptosis-related proteins cleaved caspase-3 and cleaved caspase-9, as shown by Western blot analysis. Furthermore, myricetin’s mechanism of action was associated with inhibition of PIM1 (proviral integration site for moloney murine leukemia virus), a kinase mediating transcriptional activation of genes related to cell cycle progression and cell survival [71]. In another cellular study that compared the effect of myricetin, myricetrin and quercetin on PC-3 cells viability using the MTT assay, the IC_50_ value for myricetin was 94.48 µM, which was 2–4 times lower compared to the IC_50_ of myricetrin and quercetin [72]. Another prostate cancer cellular model showed that 100 µM myricetin was associated with a 60% inhibitory growth effect [40]. While myricetin’s mechanism of action may involve inhibition of Glyoxalase 1 [45], similarly to other flavonols, myricetin may also serve as a preventive cancer agent in view of its capacity to inhibit CYP1B1, an enzyme that can metabolize polyaromatic hydrocarbons into toxic intermediates [73]. Myricetin may also synergize with chemotherapy agents as CYP1B1 is also involved in anti-cancer drug metabolism [73]. In bladder cancer cellular models, myricetin showed an IC_50_ of 72.68, 30.26, 20.94 µM in RT4, SCABER and SW780 bladder cancer cell lines, respectively [74] by MTT assay. In another cellular model that used bladder cancer T24 cell lines tested by MTT assay, myricetin induced a 2.6–61% decrease in cell viability at concentrations of 20–100 µM after 12 h, with an IC_50_ value of 85 µM for 24 h [75].

#### 3.3.2. Clinical Studies

Only a few epidemiologic studies were identified by the systematic search. In the prostate cancer cohort assessed by Geybels et al. discussed above [50], a higher myricetin consumption was also associated with a lower risk of being diagnosed with advanced prostate cancer, although overall intake was low (average intake < 1.5 mg/a day). One large cohort study estimated the flavonoid intakes of 10,054 individuals based on dietary habits and flavonoid concentrations in Finnish foods and computed incident cases of the diseases from available national public health registers. In the entire cohort, total myricetin daily intake was 0.12 mg. Of all the malignancies considered (lung, prostate, breast, urinary, colo-rectal), prostate cancer was the only tumor that was associated with myricetin consumption, with a significantly lower risk in the fourth vs. the first quartile (0.43; 95% CI: 0.22, 0.86; *p* = 0.002) and in the third vs. the first quartile (0.51; 95% CI: 0.28, 0.91) [76]. In the study by Garcia et al. [51] referred to above, no statistically significant association between myricetin consumption and bladder cancer was identified, which was consistent with the results obtained by Knekt et al. [76].

Results obtained in in vitro, animal and human studies are schematically reported in Table 2, Table 3 and Table 4, respectively.

## 4. Metabolism and Bioavailability

One of the limitations that must be considered when exploring the potential applications of naturally occurring flavonols in humans is their generally low bio-availability. Also, the optimal serum levels required to achieve the desired clinical effects are unknown for all three flavonols reviewed.

Kampferol, which is metabolized by sulphate and glucuronic acid conjugation in the liver, can be absorbed by both passive and facilitated diffusion [77]. One of the few bio-availability studies conducted to assess the pharmacokinetics of flavonols in humans, enrolled four healthy men and four healthy women (age range: 26–47 years), who were administered 9 mg kaempferol obtained from endive and observed for one day. Also, subjects were instructed not to consume any flavonoid-rich foods prior and during the study. A mean maximum plasma concentration of 0.1 mM was reported after 5.8 h, which was indicative of absorption from the colon or the distal section of the small intestine, differently from other flavonoids, such as rutin, that are absorbed from the large intestine for the greatest part. Furthermore, only 1.9% of the administered kaempferol was found in 24-h urine. Glycosylated kaempferol amounted to 14% of the total kampferol content of endive and was likely responsible for an early absorption peak that was reported in most subjects. Kaempferol-3-glucuronide was the major compound detected in plasma and urine [78]. In another small clinical study that included 8 males and 7 females (age range: 19–56 years), mean plasma concentrations of 15 ng/mL kaempferol were detected after participants ingested 27 mg kaempferol from tea, with 2.5% of the total kaempferol dose consumed detected in urine. To the best of our knowledge, no pharmacokinetic studies are available in humans for fisetin and myricetin, so pharmacokinetic data in humans can only be extrapolated from data obtained in murine models. Fisetin oral bioavailability is expected to be low because of its low aqueous solubility and its extensive first-pass metabolism [79]. In mice, after oral administration of fisetin 50 mg/kg of body weight, the fisetin parent form could be detected in serum transiently only during the absorption phase, while the peak concentration of fisetin sulfates and glucuronides was 72.1 µM [80]. In another pharmacokinetic study conducted in mice that were administered myricetin both intravenously and orally, the absolute bioavailability was found to be 9.62% and 9.74% at 50 mg/kg and 100 mg/kg of body weight, respectively, while an oral dose of 50 mg/kg of body weight yielded an average peak concentration of 4.6 µM [81].

## 5. Discussion

Our systematic search of the literature found consistent evidence derived from cellular and, in some cases, rodent models that kaempferol, fisetin and myricetin may exert anti-neoplastic activity in prostate and bladder cancer. Epidemiologic studies exploring a potential association between dietary daily intake of these individual flavonols and prostate and bladder cancer suggest that kaempferol and myricetin may be associated with a lower risk of advanced prostate cancer and all-stage prostate cancer, respectively, while total flavonol intake was associated with a reduced risk of bladder cancer in the EPIC study [21]. The attractiveness of these compounds as anti-cancer agents lies in multiple factors, including their availability on the market, low cost, low toxicity, low likelihood of pharmacological interactions as well as potential for synergism with anti-cancer medications. Our work group has reported the encouraging activity of isoquercetin against adverse events associated with sunitinib, including fatigue, hand and foot syndrome, rash in a small cohort of 12 patients with kidney cancer [24]. Furthermore, we reported an unusual complete response obtained with low dose oral cyclophosphamide and high doses of oral quercetin in an older patient with advanced urothelial carcinoma [82]. In this regard, it must be noted that isoquercetin, the glycosylated form of quercetin, is approximately 10 times more bioavailable compared to quercetin [25,83]. Similarly, it is likely that glycosylated myricetin [84] and kaempferol [78], which are commonly available in food, are more bio-available compared to their aglycone forms.

Given the findings obtained (1) in pharmacokinetic studies in humans and rats; (2) in cellular models of prostate and bladder cancer; (3) in murine models of prostate and bladder cancer; (4) in epidemiological studies, we believe that there is a sufficiently strong rationale to explore the potential clinical applications of kaempferol, myricetin and fisetin in selected patients affected by prostate and bladder cancer. We believe that prevention of recurrence is the ideal setting for initial testing, although fisetin may be specifically explored in combination with cabazitaxel after docetaxel failure [55], especially in selected patients with aggressive disease [85], on the basis of the results of the preclinical model referred to above [55]. Both bladder and prostate cancer can be treated with radical surgery if they present as organ-confined disease, with a recurrence risk in the range of 20–40% for prostate cancer after prostatectomy [86], up to 50% for bladder cancer after cystectomy [87] and up to 30–40% after TURBT [88]. Given the limited number of adjuvant systemic treatment options, a combination of kaempferol, fisetin and myricetin may be clinically tested as a nutritional approach after radical surgery for prostate and bladder cancer as an adjunct intervention in addition to standard of care. Clinical trials must be designed to compare different dose levels and assess bioavailability. Given the lack of experimental data, starting doses can be empirically hypothesized based on pharmacokinetic data and target peak concentrations extrapolated from pre-clinical models. Given the peak concentrations of 0.1 µM achieved with 9 mg of kaempferol, a target peak serum concentration of 10 µM for kaempferol may be obtained by administering 900 mg. For myricetin, a 46 µM peak concentration may be obtained by administering 500 mg/kg in rats, which are equivalent to approximately 40 mg/kg in humans. For fisetin, effective peak concentrations may be obtained by administering the equivalent of 50 mg/kg in rats, that is, 4 mg/kg in humans. We may therefore speculate that in clinical trials, daily oral kaempferol and myricetin doses may be in the range of 900–2500 mg/a day while fisetin may be effective at lower doses (250–500 mg/day). In this regard, studies conducted with isoquercetin that have measured peak serum concentrations and assessed them to target concentrations with a biological activity have set an example and represent the basis for further research in the field [83].

## 6. Conclusions

Kaempferol, fisetin and myricetin are normally ingested as they are naturally present in vegetables and fruits. They are also currently available on the global market as nutritional supplements. Available evidence shows that these compounds have potential activity against bladder and prostate cancer. Although concentrations tested in pre-clinical models are far higher than peak serum levels that can be obtained with consumption of fruit and vegetables, higher serum levels can be obtained with consumption of nutritional supplements. The expected toxicity is low, so higher daily doses in the range of grams can probably be administered to compensate for the low bioavailability. The maximum tolerated dose for kaempferol, fisetin and myricetin is yet to be established. Clinical trials must be designed not only to prove the effectiveness and safety of a nutritional intervention based on these flavonols consumed as plant-derived extracts, but also to assess the optimal dose and duration of use. Based on the evidence reviewed, both patients with active cancer and those without cancer but at high risk of recurrence/occurrence might benefit from consuming these flavonols. Further clinical research is warranted.

## Figures and Tables

**Table 1:** Included and excluded articles.

		Entries Found ^1^	Excluded			Included	
			original work, but not experimental	not original work	not involving the single pure substance or the prostate/bladder cancer	Preclinical	Clinical
Prostate cancer	Kaempferol	53	3	6	25	17	2
	Myricetin	16	2	2	5	5	2
	Fisetin	28	2	8	6	12	0
Bladder cancer	Kaempferol	13	1	4	4	4	1
	Myricetin	3	0	0	0	2	1
	Fisetin	4	0	0	1	3	0

^1^ After removal of duplicate articles.

**Table 2:** Results of in vitro models with available IC_50_.

Flavonol (Kaempferol, Fisetin, Myricetin)	Model (Prostate vs. Bladder Cancers)	Cell Line	Assay	IC_50 (uM)_	Reference
Kaempferol	Prostate cancer	LNCaP	MTT assay	28.8 ± 1.5 μM (with 1 nM DHT)	[33]
		PC-3		58.3 ± 3.5 μM (with 1 nM DHT)	
		RWPE-1		69.1 ± 1.2 μM (with 1 nM DHT)	
		DU-145	Cell count with Trypan Blue	38.35 ± 1.94 μM	[34]
		PC-3		33.29 ± 2.96 μM	
		LNCaP	WST-1 assay	29 ± 6 μM	[35]
		DU-145	MTT assay	50 ± 0.00 μM	[36]
		PC-3	WST-1 assay	1.8 uM	[37]
	Bladder cancer	EJ	MTT assay	54.7 μM	[38]
		EJ	CCK-8 assay	78.4 μM (T24 h) 38.1 μM (T48 h)	[47]
		T24		85.3 μM (T24 h) 54.2 μM (T48 h)	
Fisetin	Prostate cancer	PC-3	WST-1 assay	>50 μM	[52]
		DU-145		>50 μM	
		LNCaP		34.1 ± 7.7 μM	
		LNCaP	CyQuant cell proliferation assay	22.65 μM	[62]
		PC-3		32.50 μM	
Myricetin	Prostate cancer	PC-3	CCK-8 assay	47.6 μM	[68]
		DU-145		55.3 μM	
		C4-2		79.9 μM	
		RWPE1		362.1 μM	
		PC-3	MTT assay	94.48 μM	[70]
	Bladder cancer	SV-HUC SW-780	CellTiterGlo reagent assay	>200 μM 20.9 μM	[72]
		T24	MTT assay	85 μM	[73]

**Table 3:** Results of animal models.

Flavonol (Kaempferol, Fisetin, Myricetin)	Model (Prostate vs. Bladder Cancer)	Cell Line	Dose	Results	References
Kaempferol	Bladder cancer	5637	50, 100, 150 mg/kg every day for 4 weeks	Tumor growth and metastasis suppression	[39]
		T24	150 mg/kg every day for 31 days	Tumor growth inhibition Tumor volume: control mice (≃3000 mm^3^) vs. Tumor volume in treated mice (≃1000 mm^3^) DNA methylation modulation by inhibiting DNMT3B	[47]
Fisetin	Prostate cancer	NB26	1 mg/kg twice weekly for 28 days	Epithelial-to-mesenchymal transition inhibition	[53]
		CWR22Rυ1	1 mg/animal twice weekly for 46 days	Tumor growth inhibition Tumor reached a volume of 1200 mm^3^ after 26 days in control mice and after 46 days in treated mice-PSA secretion inhibition	[54]
		22Rν1	20 mg/kg; 3 times/week for 7 weeks	Tumor growth Inhibition by decreasing proliferation and inducing apoptosis Tumor volume: control mice (≃1800 mm^3^) vs. Tumor volume in treated mice (≃1300 mm^3^) Overall survival increase	[55]
		PC-3M-luc-6		Tumor growth inhibition Tumor volume: control mice (≃600 mm^3^) vs. Tumor volume in treated mice (≃500 mm^3^)—Metastasis inhibition	
		NB11 NB26	40 mg/kg~1 mg/animal) twice weekly until tumors reached a volume of 1200 mm^3^	Synthesis and degradation inhibition of hyaluronan, an enzyme involved in cancer progression	[56]
	Bladder cancer	Rat model of bladder cancer induced by intravesical N-methyl-N-nitrosourea	200 mg/kg weekly for 18 weeks	Apoptosis induction	[66]
Myricetin	Prostate cancer	PC-3	25 mg/kg every 2 days for 40 days	Cancer growth inhibition Tumor volume: control mice (≃1800 mm^3^) vs. Tumor volume in treated mice (≃600 mm^3^)-Epithelial-to-mesenchymal transition inhibition	[68]

**Table 4:** Results of human studies.

Flavonol (Kaempferol, Fisetin, Myricetin)	Prostate vs. Bladder Cancer	Total Sample Size	Estimated Daily Intake (Mean)			Results (Report p)	References
Kaempferol	Prostate cancer	433 men with primary, histologically confirmed prostate cancer and 538 population-based controls	µg/day	OR (95% CI)	OR (95% CI) Further adjusted for vegetable intake.	Cancer risk reduction	[49]
			<1447.5	1.00	1.00		
			1447.5–2990.5	0.90 (0.63–1.27)	0.90 (0.63–1.28)		
			2990.5–6056.8	0.73 (0.51–1.04)	0.74 (0.52–1.07)		
			>6056.8	0.83 (0.58–1.18)	0.85 (0.59–1.22)		
		3362 prostate cancer patients	6.5 (4.4–9.4) mg/day Hazard ratios of stage IV prostate cancer for the highest versus the lowest quartile of intake of kaempferol: 0.78 (95% CI: 0.61, 1.00;)			Dietary intake was not associated with overall or nonadvanced prostate cancer risk; decreased risk of advanced (stage III/IV) or stage IV prostate cancer.	[50]
	Bladder cancer	Cases(*n* = 495)	0.97 ± 1.15 mg/day			Intake of kaempferol is not protective against bladder cancer risk	[53]
		Controls (*n* = 1112)	1.03 ± 1.18 mg/day				
Myricetin	Prostate cancer	3362 prostate cancer patients	1.4 mg/day (0.9–2.0) Hazard ratios of stage IV prostate cancer for the highest versus the lowest quartile of intake of myricetin: 0.71 (95% CI: 0.55, 0.91).			Dietary intake was not associated with overall or nonadvanced prostate cancer risk; decreased risk of advanced (stage III/IV) or stage IV prostate cancer.	[50]
	Bladder cancer	Cases (*n* = 495)	0.23 ± 0.35 mg/day 0.21 ± 0.34 mg/day			Intake of myricetin is not protective against bladder cancer risk.	[51]
		Controls (*n* = 1112)					

## Data Availability

No new data were created or analyzed in this study. Data sharing is not applicable to this article
